# Fever is associated with earlier antibiotic onset and reduced mortality in patients with sepsis admitted to the ICU

**DOI:** 10.1038/s41598-021-03296-7

**Published:** 2021-12-14

**Authors:** Aileen Dias, Vitoria Campanha Gomez, Luciana Rosa Viola, Anna Carolina Pedrazani Rodrigues, Stefanie Piber Weber, Luiza Tartaro, Leonardo da Silva Marques, Márcio Manozzo Boniatti

**Affiliations:** 1 Universidade La Salle, Victor Barreto Avenue, 2288, Canoas, 92010-000 Brazil; 2 Universidade do Vale do Rio dos Sinos, São Leopoldo, Brazil; 3 Department of Critical Care, Hospital de Clínicas de Porto Alegre, Porto Alegre, Brazil

**Keywords:** Infectious diseases, Fever

## Abstract

To evaluate the association of body temperature with mortality in septic patients admitted to the ICU from the ward. In addition, we intend to investigate whether the timing of antibiotic administration was different between febrile and afebrile patients and whether this difference contributed to mortality. This is a retrospective cohort study that included sepsis patients admitted to the ICU from the ward between July 2017 and July 2019. Antibiotic administration was defined as the initiation of antimicrobial treatment or the expansion of the antimicrobial spectrum within 48 h prior to admission to the ICU. Regarding vital signs, the most altered vital sign in the 48 h prior to admission to the ICU was considered. Two hundred and eight patients were included in the final analysis. Antibiotic administration occurred earlier in patients with fever than in patients without fever. Antibiotic administration occurred before admission to the ICU in 27 (90.0%) patients with fever and in 101 (64.7%) patients without fever (p = 0.006). The mortality rate in the ICU was 88 in 176 (50.0%; 95% CI 42.5–57.5%) patients without fever and 7 in 32 (21.9%; 95% CI 6.7–37.0%) patients with fever (p = 0.004). In the multivariate analysis, absence of fever significantly increased the risk of ICU mortality (OR 3.462; 95% CI 1.293–9.272). We found an inverse association between body temperature and mortality in patients with sepsis admitted to the ICU from the ward. Although antibiotic administration was earlier in patients with fever and precocity was associated with reduced mortality, the time of antibiotic administration did not fully explain the lower mortality in these patients.

## Introduction

Rapid recognition and timely treatment are important in the management of sepsis^1^. In this scenario, the presence of fever increases the suspicion of infection, contributing to earlier recognition^2^. However, many patients with sepsis do not have a fever, making identification more difficult^3^. The absence of fever can lead to a delay in diagnosis and interventions, especially in the administration of antibiotics, and may contribute to increased mortality in afebrile patients.

In addition to the contribution to identification, several potential benefits of increasing body temperature when an infectious insult is present have been described, such as negative feedback on the release of pyrogenic cytokines^4^, improved antibiotic activity^5^ and immune cell function^6,7^. Several studies have demonstrated this association of fever with lower mortality in patients with infection in the emergency room^8–12^ and in the intensive care unit (ICU)^11,13,14^. However, most of these studies did not assess the impact of early interventions^11–14^. The studies that controlled for quality-of-care measures included only patients from the emergency room^8–10^. In the present study, we evaluated the association of body temperature with mortality in septic patients admitted to the ICU from the ward. In addition, we investigated whether the timing of antibiotic administration was different between febrile and afebrile patients and whether this difference contributed to mortality.

## Methods

This is a retrospective cohort study. We conducted a secondary analysis of prospectively collected data that included sepsis patients admitted to the ICU from the ward between July 2017 and July 2019. The study was conducted at the Hospital de Clínicas de Porto Alegre (HCPA). HCPA is a tertiary hospital, with approximately 30,000 admissions per year. The ICU comprises 33 clinical and surgical beds. The study was approved by the research ethics committee of the HCPA and the informed consent form was waived by this ethics committee due to the retrospective nature of the study. All methods were performed in accordance with the Declarations of Helsinki.

The study included adult patients with sepsis admitted to the ICU from the ward. Sepsis was defined according to the Sepsis-3 criteria based on the identification of organ dysfunction caused by an unregulated host response to infection. For this definition, an increase of 2 points or more was used in the Sequential Organ Failure Assessment (SOFA) score^15^. Septic shock was defined as the need for a vasopressor to maintain an average blood pressure of 65 mmHg or more. For this definition, we did not include the requirement for lactate above 2 mmol/L. For patients with more than one ICU admission during the study period, only the first admission was considered.

Two collection methods were used: a review of a prospectively constructed database and a review of electronic medical records. Demographic and clinical data, as well as the outcomes of interest, were collected prospectively. The following variables were included: age, sex, Charlson's index, Simplified Acute Physiology Score (SAPS) 3 and SOFA score on admission to the ICU, focus of infection, length of stay in the hospital prior to admission to the ICU, presence of septic shock, length of stay in the ICU and in the hospital and mortality in the ICU and in the hospital. The variables collected from the review of electronic medical records were as follows: time between admission to the ICU and administration of antibiotics and vital signs recorded in the 48 h prior to admission to the ICU. Antibiotic administration was defined as the initiation of antimicrobial treatment or the expansion of the antimicrobial spectrum within 48 h prior to admission to the ICU. Regarding vital signs, the most altered vital sign in the 48 h prior to admission to the ICU (higher body temperature, higher heart rate, higher respiratory rate, lower systolic blood pressure and lower peripheral oxygen saturation) was considered. Fever and hypothermia were defined as body temperature ≥ 38.0 °C and ≤ 36.0 °C, respectively, in the 48 h prior to admission to the ICU.

### Statistical analysis

The normality of continuous variables was assessed using the Kolmogorov–Smirnov test. Continuous variables are expressed as the mean ± SD or median and interquartile range. Categorical variables are presented as absolute numbers and percentages. Student’s t or Mann–Whitney tests were used for continuous variables, and a chi-squared test was used for categorical variables. Variables with associated with ICU mortality with a p-value < 0.20 in the univariate analysis were included in the logistic regression model. Final model was built using a stepwise backward process. As the delay in antibiotic administration can contribute to the mortality of afebrile patients, we evaluated the relationship between the time of antibiotic administration and the mortality of afebrile patients using Student's t-test. In addition, the interaction term time to onset of antibiotic*afebrile was added to the initial logistic regression model to assess the degree of contribution of delay in antibiotic onset time to excess mortality experienced by afebrile patients. The results are presented as odds ratios (ORs) and 95% confidence intervals. A value of p < 0.05 was considered statistically significant. The statistical analysis was performed using SPSS software version 20.0.

## Results

**Figure 1:**
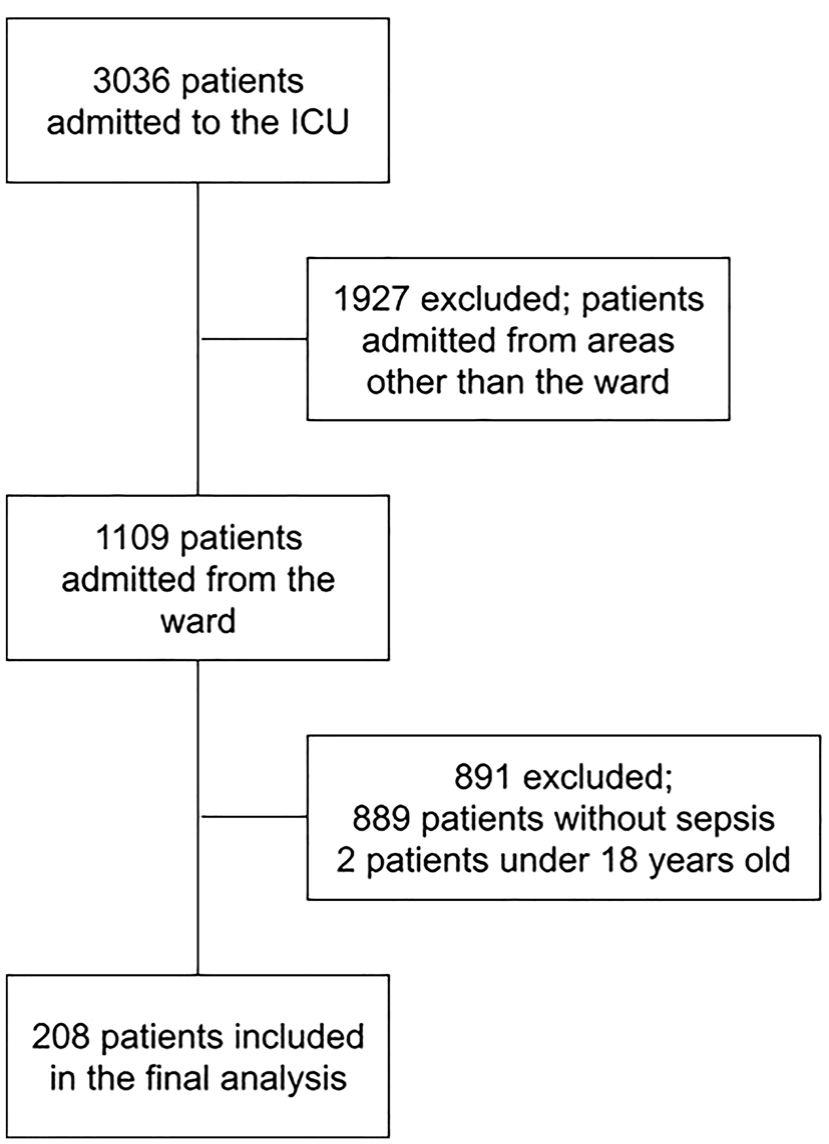
Flowchart of patients inclusion.

During the study period, 3036 patients were admitted to the ICU. Of these, 208 patients were included in the final analysis (Fig. 1). Only 32 (15.4%) patients had fever in the 48 h prior to admission to the ICU. The clinical and demographic characteristics of the patients are described in Table 1. Patients without fever had a higher SOFA score on admission to the ICU.

**Table 1:** Univariate analysis comparing febrile and afebrile patients with sepsis admitted to an Intensive Care Unit.

	Afebrile (n = 176)	Febrile (n = 32)	p
Sex, male, n (%)	103 (58.5)	19 (59.4)	0.93
Age, years	66.0 (55.3–76.0)	59.0 (47.3–72.8)	0.10
SAPS 3	75.5 ± 13.0	72.9 ± 12.8	0.30
SOFA	7.0 (4.3–10.0)	5.0 (3.0–8.0)	0.008
Charlson index	2.5 (2.0–5.0)	2.0 (1.3–4.8)	0.96
**Focus of infection, n (%)**			0.72
Skin/soft tissue	11 (6.2)	2 (6.2)	
Bloodstream infection	8 (4.5)	2 (6.2)	
Urinary	9 (5.1)	3 (9.4)	
Pulmonary	75 (42.6)	12 (37.5)	
Abdominal	37 (21.0)	7 (21.9)	
Unknown origin	29 (16.5)	4 (12.5)	
Other	7 (4.0)	2 (6.2)	
Septic shock, n (%)	106 (60.2)	17 (53.1)	0.45
Length of hospital stay before ICU admission, days	13.0 (6.0–23.3)	14.0 (7.3–24.0)	0.66
Systolic arterial pressure, mmHg	93.0 (83.3–109.0)	89.5 (78.0–102.0)	0.14
Heart rate, beats/min	110.0 (99.3–123.8)	124 (115.5–135.8)	< 0.001
Respiratory rate, breath/min	23.0 (20.0–25.0)	24.0 (22.0–29.8)	0.004
Oxygen saturation, %	91.0 (87.0–94.0)	90.5 (84.5–92.8)	0.29
Time between admission to the ICU and antibiotic administration, hours	− 3.0 (− 14.0–0.0)	− 9.0 (− 24.5 to − 2.75)	0.04
Length of ICU stay, days	4.0 (2.0–10.0)	4.0 (2.0–9.8)	0.77
Length of hospital stay, days	30.0 (15.0–47.0)	39.0 (24.0–55.0)	0.09
ICU mortality, n (%)	88 (50.0)	7 (21.9)	0.004
Hospital mortality, n (%)	116 (65.9)	15 (46.9)	0.04

Antibiotics were administered in the 48 h prior to admission or on the day of admission to the ICU in 186 (89.4%) patients. Antibiotic administration occurred prior to admission, at the time of admission or after the first hour of admission to the ICU in 128 (68.8%), 50 (26.9%) and 8 (4.3%) patients, respectively. Patients with fever received antibiotics earlier than patients without fever (Table 1). Antibiotic administration occurred before admission to the ICU in 27 (90.0%) patients with fever and in 101 (64.7%) patients without fever (p = 0.006). The ICU mortality between patients who received antibiotics before ICU admission and patients who received antibiotics at the time or after ICU admission were 39.8% and 53.4% (p = 0.08), respectively.

The mortality rate in the ICU was 88 in 176 (50.0%; 95% CI 42.5–57.5%) patients without fever and 7 in 32 (21.9%; 95% CI 6.7–37.0%) patients with fever (p = 0.004) (Table 1). In the multivariate analysis, absence of fever significantly increased the risk of ICU mortality (OR 3.462; CI 95% 1.293–9.272) (Table 2). We examined the association of antibiotic administration time with ICU mortality in afebrile patients only. No difference was found in the time of antibiotic administration between the afebrile patients who died (− 3.0; − 11.5 to 0.0) and those who survived (− 4.0; − 16.7 to 0.0) (p = 0.19). When the interaction term time to onset of antibiotic*afebrile was added to the initial logistic regression model, the interaction term was not significant. We only had 12 patients with hypothermia. Even after excluding these patients, the presence of fever remained associated with mortality. In the multivariate analysis of factors associated with hospital mortality, only SAPS 3 (OR 1.065; p < 0.001) and septic shock on admission (OR 2.083; p = 0.028) maintained an independent association.

**Table 2:** Multivariate analysis of the factors associated with ICU mortality.

Variable	OR	95% CI	p
SAPS 3	1.040	1.011–1.069	0.006
Afebril	3.462	1.293–9.272	0.013
Time of antibiotic administration	1.022	1.001–1.044	0.044

Hosmer–Lemeshow chi-squared = 7.119, p = 0.524. The independent variables included were age, SAPS 3, septic shock, afebril and time of antibiotic administration.

## Discussion

We found that afebrile patients with sepsis admitted to the ICU from the ward had higher mortality than febrile patients. Afebrile patients had a 28.1% higher absolute risk of ICU mortality. The association of the absence of fever with mortality persisted after adjusting for several confounding variables. In addition, afebrile patients took longer to receive antibiotics. Although this delay potentially contributed to the excess mortality, it does not fully explain the difference found. To the best of our knowledge, this is the first study to evaluate the association between body temperature and mortality in patients with sepsis admitted to the ICU from the ward while considering the impact of antibiotic administration.

There is some biological plausibility to explain the association between fever and survival in patients with sepsis. Experimental studies have already suggested that fever, in an infectious disease scenario, can be a beneficial physiological response^16,17^. The increase in body temperature can provide negative feedback in the secretion of pyrogenic cytokines, inhibit the replication of bacteria and viruses, improve the effect of antibiotics and improve the function of immune cells^5–7^. On the other hand, the inability to increase body temperature in response to an infectious insult can be a physiological indicator of a more severe patient or a weaker immune response. The association between nonelevated temperature and mortality has been previously demonstrated in patients with sepsis in the emergency room^8–12^ and in the ICU^13,14^. However, most of these studies did not verify the impact of antibiotic administration on mortality. In studies that controlled for quality-of-care measures, the association of fever with lower mortality remained. Henning et al. found an absolute risk of in-hospital mortality 21% higher in afebrile patients^8^. Sundén-Cullberg et al., in a cohort that included patients admitted to the ICU from the emergency room with severe sepsis or septic shock, found a 5% decrease in in-hospital mortality for each 1 °C increase in body temperature^9^, and the results were confirmed recently after adjusting these data for disease severity measured by SAPS 3^10^. Our study reinforces this association between body temperature and mortality in patients with sepsis by confirming these results in a cohort of patients admitted to the ICU from the ward and after adjusting for several confounding variables.

Another important point to be discussed is the quality of care offered to patients with fever in relation to afebrile patients. Febrile patients receive antibiotics more frequently and earlier and receive a higher volume in the first hour^8,9^. However, in these two studies, the best care did not explain the association between fever and mortality, as the absence of fever remained a predictor of mortality even after adjusting for quality-of-care measures. Our findings in the ward setting corroborate these results. Febrile patients received antibiotics earlier, and the timing of antibiotic administration was associated with mortality. However, in our analysis, the absence of fever remained a predictor of mortality after adjusting for the antibiotic administration time. In addition, in the stratified analysis, no difference was found in the time of antibiotic administration between afebrile patients who died compared with those who did not die. Afebrile patients are at a higher risk of death regardless of the quality of care, although they may suffer a second insult for receiving less antibiotics or receiving it later due to the lack of recognition of sepsis in this subgroup.

Our study has some limitations. First, the study was retrospective, with a small number of patients and in a single center, giving an inherent bias to this type of design and limiting the generalization of the results. Second, our definition of septic shock was based on the need for vasopressors, without the need for hyperlactatemia as provided in the Sepsis-3 criteria. In addition, we did not record the time of vasopressor onset to assess the time between septic shock diagnosis and antibiotic initiation. However, as the use of vasopressors in the ward is not allowed in our institution, the time of diagnosis of septic shock is very close to the time of admission to the ICU. Finally, we did not evaluate the adequacy of the antibiotics administered or other measures of quality of care. It is possible that the temperature-mortality association is related to other differences in treatment not assessed in the present study. In particular, the adequacy of empirical treatment is a very important factor in the prognosis of patients with sepsis, and this data would make the work more robust. Unfortunately, we do not have this data.

We found an inverse association between body temperature and mortality in patients with sepsis admitted to the ICU from the ward. Although antibiotic administration was earlier in patients with fever and precocity was associated with reduced mortality, the time of antibiotic administration did not fully explain the lower mortality in these patients. New studies should investigate the difference in the immune response of afebrile patients in addition to investigating ways to improve the recognition of sepsis in this subset of patients.

## References

[1] Dellinger RP, Schorr CA, Levy MM (2017). A users' guide to the 2016 surviving sepsis guidelines. Crit. Care Med..

[2] O'Grady NP, Barie PS, Bartlett JG (2008). Guidelines for evaluation of new fever in critically ill adult patients: 2008 update from the American College of Critical Care Medicine and the Infectious Diseases Society of America. Crit. Care Med..

[3] Kaukonen KM, Bailey M, Pilcher D (2015). Systemic inflammatory response syndrome criteria in defining severe sepsis. N. Engl. J. Med..

[4] Bota DP, Ferreira FL, Mélot C (2004). Body temperature alterations in the critically ill. Intensive Care Med..

[5] Mackowiak PA, Marling-Cason M, Cohen RL (1982). Effects of temperature on antimicrobial susceptibility of bacteria. J. Infect. Dis..

[6] Mace TA, Zhong L, Kilpatrick C (2011). Differentiation of CD8+ T cells into effector cells is enhanced by physiological range hyperthermia. J. Leukoc. Biol..

[7] Evans SS, Repasky EA, Fisher DT (2015). Fever and the thermal regulation of immunity: The immune system feels the heat. Nat. Rev. Immunol..

[8] Henning DJ, Carey JR, Oedorf K (2017). The absence of fever is associated with higher mortality and decreased antibiotic and IV fluid administration in emergency department patients with suspected septic shock. Crit. Care Med..

[9] Sundén-Cullberg J, Rylance R, Svefors J (2017). Fever in the emergency department predicts survival of patients with severe sepsis and septic shock admitted to the ICU. Crit. Care Med..

[10] Inghammar M, Sunden-Cullberg J (2020). Prognostic significance of body temperature in the emergency department vs the ICU in patients with severe sepsis or septic shock: A nationwide cohort study. PLoS ONE.

[11] Stoneking LR, Winkler JP, DeLuca LA (2015). Physician documentation of sepsis syndrome is associated with more aggressive treatment. West J. Emerg. Med..

[12] Drumheller BC, Agarwal A, Mikkelsen ME (2016). Risk factors for mortality despite early protocolized resuscitation for severe sepsis and septic shock in the emergency department. J. Crit. Care.

[13] Young PJ, Saxena M, Beasley R (2012). Early peak temperature and mortality in critically ill patients with or without infection. Intensive Care Med..

[14] Kushimoto S, Gando S, Saitoh D (2013). The impact of body temperature abnormalities on the disease severity and outcome in patients with severe sepsis: An analysis from a multicenter, prospective survey of severe sepsis. Crit. Care.

[15] Singer M, Deutschman CS, Seymour CW (2016). The third international consensus definitions for sepsis and septic shock (sepsis-3). JAMA.

[16] Kluger MJ (2015). Fever: Its Biology, Evolution, and Function.

[17] Mackowiak PA (1994). Fever: Blessing or curse? A unifying hypothesis. Ann. Intern. Med..

